# Lateral frontal galeal-cutaneous flap for reconstruction after orbital exenteration for advanced periorbital skin cancer

**DOI:** 10.3906/sag-1809-170

**Published:** 2021-02-26

**Authors:** Predrag KOVACEVIC, Jasmina DJORDJEVIC-JOCIC, Milan RADOJKOVIC

**Affiliations:** 1 Clinic for Plastic and Reconstructive Surgery, Faculty of Medicine, University Nis, Nis Serbia; 2 Clinic for Ophthalmology, Faculty of Medicine, University Nis, Nis Serbia; 3 Clinic for Surgery, Faculty of Medicine, University Nis, Nis Serbia

**Keywords:** Orbital evisceration, reconstructive surgery, galeal skin island flap

## Abstract

**Background/aim:**

Orbital exenteration (OE) is one of the most disfiguring procedures leading to significant deformity. Defect reconstruction is challenging, especially in elderly patients. Herein, experiences with orbital exenteration and primary reconstruction with lateral frontal galeal-cutaneous flap based on superficial temporal artery were reviewed.

**Materials and methods:**

Data on patients treated for nonmelanoma skin cancer invading the orbit during a 10-year period were analyzed. The patient demographics, tumor features, reconstructive techniques used, complications, and survival were recorded with a median follow-up of 27.5 months.

**Results:**

Included in the study were 26 patients in whom OE was performed, comprising 14 males and 12 females, with a mean age of 75.29 years (range: 61–87). The majority of the patients were treated for basal cell carcinoma with medial cantus as the primary site. All of the defects were closed using a lateral frontal galeal-cutaneous flap based on the superficial temporal artery, and in 2 patients, a temporalis muscle pedicle flap was used as an additional flap for reconstruction of the orbital roof in order to separate the brain from the empty orbit, and it was then covered with the same galeal-cutaneous flap. In 19 patients, the frontal area was closed primarily, and in 7 patients, skin graft was used for the secondary defect. There was no flap loss. Tumor-related death was registered in 3 patients (inoperable recurrent tumors) (11.5%), 7 died from complications that were unrelated to the tumors (2 were operated for recurrent orbital tumors), and 16 survived.

**Conclusion:**

The preferred method for reconstruction after OE at our university affiliated center is lateral frontal galeal-cutaneous flap based on the superficial temporal artery. Flap harvesting is simple, safe, and obtains enough tissue to cover the defects, even after extended exenteration. The complication rate is low. The simultaneous use of this flap with pedicle temporalis muscle flap is suggested only for reconstruction of the scull base after anterior cranial fossa resection.

## 1. Introduction

Advanced periorbital skin tumors sometimes have a fungating appearance and invade the orbit. In general, clinical presentation of orbital invasion is defined by visible or palpable mass, a tumor fixed to orbital wall, limited ocular motility, globe displacement, ptosis, and epiphora [1]. Older patients referred to the tumor board for larger tumors, multiple previous recurrences, and an aggressive histologic subtype (basosquamous carcinoma) should be considered for computed tomography (CT) imaging [1]. The aim of surgery is to obtain a tumor-free state, which can be reached only by orbital exenteration (OE) [1]. Although OE provides quality of life improvement by the reduction of pain, this procedure also results in devastating functional, aesthetic, psychological, and functional losses. The OE leads to loss of binocular vision (impairment of visual functions) and psychological disturbances [2]. OE is a mutilating and disfiguring procedure that typically involves the removal of the entire content of the orbit, including the periorbita, appendages, and sometimes the eyelids and a variable amount of surrounding skin. Extended OE includes the removal of the adjacent bone [3]. Most frequently, anterior ethmoidectomy and/or suprastructure maxillectomy is described [4]. After extended OE, the orbital cavity may communicate with the paranasal sinuses, nasal cavity, oral cavity, or both. [2]. The defect after OE and maxillectomy is defined as complex with communication of the oral and nasal cavity with the exterior. In such cases, some researchers have advised a rectus abdominis microvascular flap [4,5]. Thus far, OE has not been shown to provide a cure in most of the cases. On the other hand, it has been proven to control local disease, and may prolong life, especially when combined with other adjuvant therapies (chemotherapy and radiotherapy).

In general, OE is used to treat tumors, and rarely, inflammation and trauma. The most common indication for OE is a periocular malignant tumor invading the orbit and orbital wall. Absolute indications for OE are the invasion of the muscles and the fat tissue of the orbital apex, and infiltration of the conjunctiva and/or sclera. Although basal cell carcinoma is the most common malignancy in the periorbital region (90%), only 0.8% to 5.5% of these tumors invade the orbit [1]. The majority of exenterations are performed for tumors originating from orbital structures, and periorbital malignancies, such as the eyelids or periocular skin. Most of skin tumors invading orbit are squamous cell carcinoma, followed by basal cell carcinomas, sebaceous-gland carcinomas, and melanomas [2,6,7]. Meyer and Zaoli classified OE for tumors in relation to the extent of destruction involved in the surgery into 4 types:

–Type I: the palpebral skin and conjunctiva are spared,

–Type II: only the palpebral skin is spared (orbital contents and conjunctiva are removed),

–Type III: both eyelids are removed with the orbital contents, 

–Type IV: the eyeball, eyelids, and appendages of the eye are removed with the involved bone structures [8]. 

The most important decision after OE is whether to perform primary or secondary reconstruction. Some facts must be kept in mind. Early recurrence can be easily recognized when the cavity is exposed for secondary reconstruction; therefore, the spontaneous granulation technique is used in cases in which complete tumor clearance is uncertain. However, some case series have reported a high rate of sino-orbital fistula following spontaneous granulation. Skin graft reconstruction has been shown to speed up the healing process. In order to separate the orbit from the surrounding cavities, and produce an acceptable aesthetic outcome, several methods are to be considered for lining the orbit (skin grafts or local flaps) or filling it, in order to exclude nasal cavity or protect the brain when the orbital roof is resected [2]. Regional flaps (temporalis muscle, midline forehead, frontal, or temporo-parietal fascial flaps, and dermis-fat graft) and microsurgical flaps (rectus abdominis, latissimus dorsi, radial forearm, and lateral arm flaps) could be used to reconstruct the cavity. Only regional muscle flaps, rather than fascia, has shown better results, by providing better nutrition to the skin graft [2]. When the resection of the dura, and its reconstruction, are performed, in addition to OE, primary reconstruction is mandatory, in order to avoid the risk of meningitis. The flap used in this study was a lateral frontal galeal-cutaneous flap based on the superficial temporal artery.

## 2. Materials and methods

The data from medical records of 26 consecutive patients, in whom OE for skin cancer invading the orbit was performed during the period from January 2005 to December 2014, were retrospectively analyzed. On admission, informed consent for the treatment and data of the scientific analysis were obtained from all of the patients included in the study. Additional informed consent was obtained from all of the individual participants for whom photos were included in this article. Institutional Ethical Committee approval was obtained before undertaking the study. Demographic data (sex, age), tumor site and histopathology, previous treatment, type of reconstruction used, and complication rate, as well as operative time, hospital stay, recurrence rate, and cause of death were recorded. The disease stage for each case was assigned according to American Joint Committee of Cancer as T4a (advanced infiltrative, but thought to be resectable). In this study, OE was performed as the sole procedure (Meyer and Zaoli Type III) or as extended OE with resection of one or more of the bony walls (Meyer and Zaoli Type IV). In all of the patients, the reconstructive procedure was primarily performed immediately after exenteration. The lateral frontal flap was raised during surgery and transferred to the defect. The flap consisted of skin, subcutaneous tissue, and galea in the lateral frontal region based on the superficial temporal artery. Only the periostea were left in place. The donor site was closed primarily, or skin grafted. In cases of extended exenteration with the resection of orbital roof, the temporalis muscle pedicle flap was transposed first to separate the cranial contents from the orbit, followed by the galea-cutaneous frontalis flap.

### 2.1. Statistical analysis 

The following statistical parameters were presented by the descriptive statistical analysis: arithmetic mean, standard deviation, absolute frequency (N), and structure index (%).

## 3. Results 

The demographic characteristics, primary site of the tumors and pathological characteristics of study the participants are shown in Table 1.

**Table 1:** Sex and age distribution, primary site of tumors requiring OE, and tumor histology.

		No.	%
Sex	F	12	46.15
	M	14	53.85
Age (years)	61–70	5	19.23
x̄ ± SD = 75.69 ± 6.28	70–80	12	46.15
	81+	9	34.62
Total		26	100
Primary site		No.	%
Medial canthus		13	50.00
Lower lid		9	34.62
Upper lid		2	7.69
Lateral canthus		2	7.69
Total		26	100
Pathology		No.	%
Basal cell skin cancer		15	57.69
Squamous cell skin cancer		11	42.31
Total		26	100

Herein, 14 males and 12 females, with a mean age of 75.69 + 6.28 years (range: 61–87), underwent surgery.

The most common primary origin was the medial cantus region 13 (50%). The orbital roof and lateral part of supraorbital arch (anterior cranial fossa) were removed, exposing the dura, but without dura resection in 2 cases. In 4 surgeries, a suprastructure maxillectomy was performed.

Tumor histopathology included the following: basal cell carcinoma (BCC) in 15 (57.69%) patients and squamous cell carcinoma (SCC) in 11 (42.31%) patients. In the BCC group, the incidence of infiltrative, basosquamous, and nodular BCC was recorded in 8, 4, and 3 patients, respectively. The orbital exenteration extent and reconstruction after exenteration are shown in Table 2.

**Table 2:** OE extent and reconstruction after exenteration, and reconstruction after exenteration.

Types of resection	Bone(s) removed	No.	%
Exenteration type III	None	14	53.85
Exenteration type IV	Ethmoid	6	23.07
	Maxilla, malar	3	11.53
	Frontal, malar	2	7.70
	Ethmoidal, maxilla, malar	1	3.85
Total		26	100
Flap		No.	%
Lateral frontal galeal -cutaneous		24	92.31
Lateral frontal galeal -cutaneous and temporalis muscle		2	7.69
Total		26	100

In 11 (42.30%) patients, the tumor was primary advanced, without previous treatment, and in 15 (57.7%) patients, a recurrent cancer of periorbital skin that infiltrated the orbit was diagnosed. The number of previous treatments ranged from 1 to 3. According to the classification of Meyer and Zaoli, in 14 (53.84%) patients, type III exenteration was performed (both eyelids were removed with the orbital contents), and in 12 patients, 1 or more of the orbital walls were removed (Type IV: the eyeball, eyelids, and appendages of the eye were removed with the involved bone structures). Ethmoidectomy was performed most dominantly. Clear margins after exenteration were recorded in 20 patients (76.92%), while in 6 patients (23.08%), the margins were positive. All defects after exenteration were reconstructed using local flaps. In all of the patients, the lateral frontal galeal-cutaneous flaps based on superficial temporal arteries were harvested for primary reconstruction. In 2 patients who underwent anterior cranial fossa resection, lateral frontal galeal-cutaneous flaps were raised, followed by harvesting and transposition of a pedicle temporalis muscle flaps in the orbit through the lateral orbital wall in order to separate the cranial content from the orbit. After temporalis flap positioning and fixation, the reconstructions were completed by transposition of the lateral frontal galeal-cutaneous flaps. Donor regions of the lateral frontal flaps were closed primarily in 19 patients (73%) and in 7 patients, partial thickness skin grafts were used. In all of the patients, after resection of the orbital floor (maxillectomy), a trans-nasal maxillary sinus tamponade was used for 5 days. 

**Figure 1:**
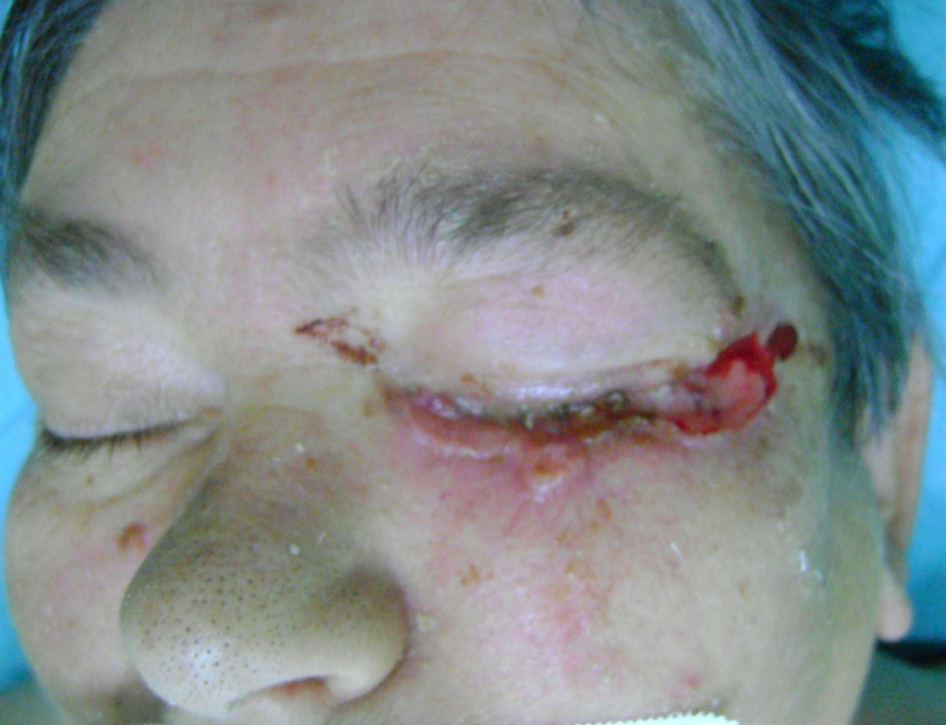
Basal cell skin cancer of lower lid.

**Figure 2:**
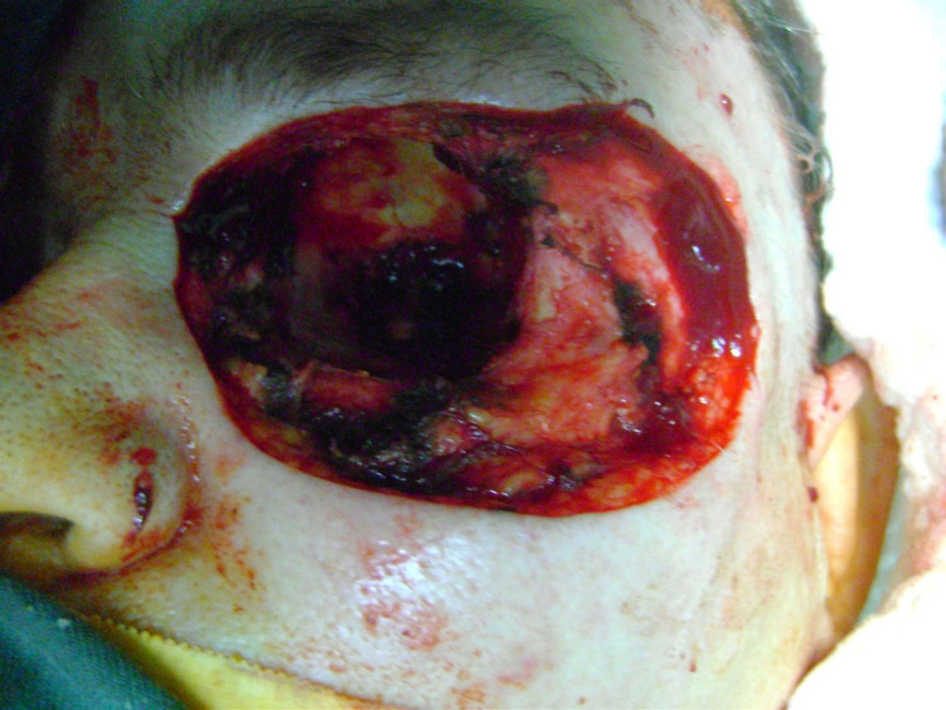
Operative defect including the OE and suprastructure maxillectomy.

**Figure 3:**
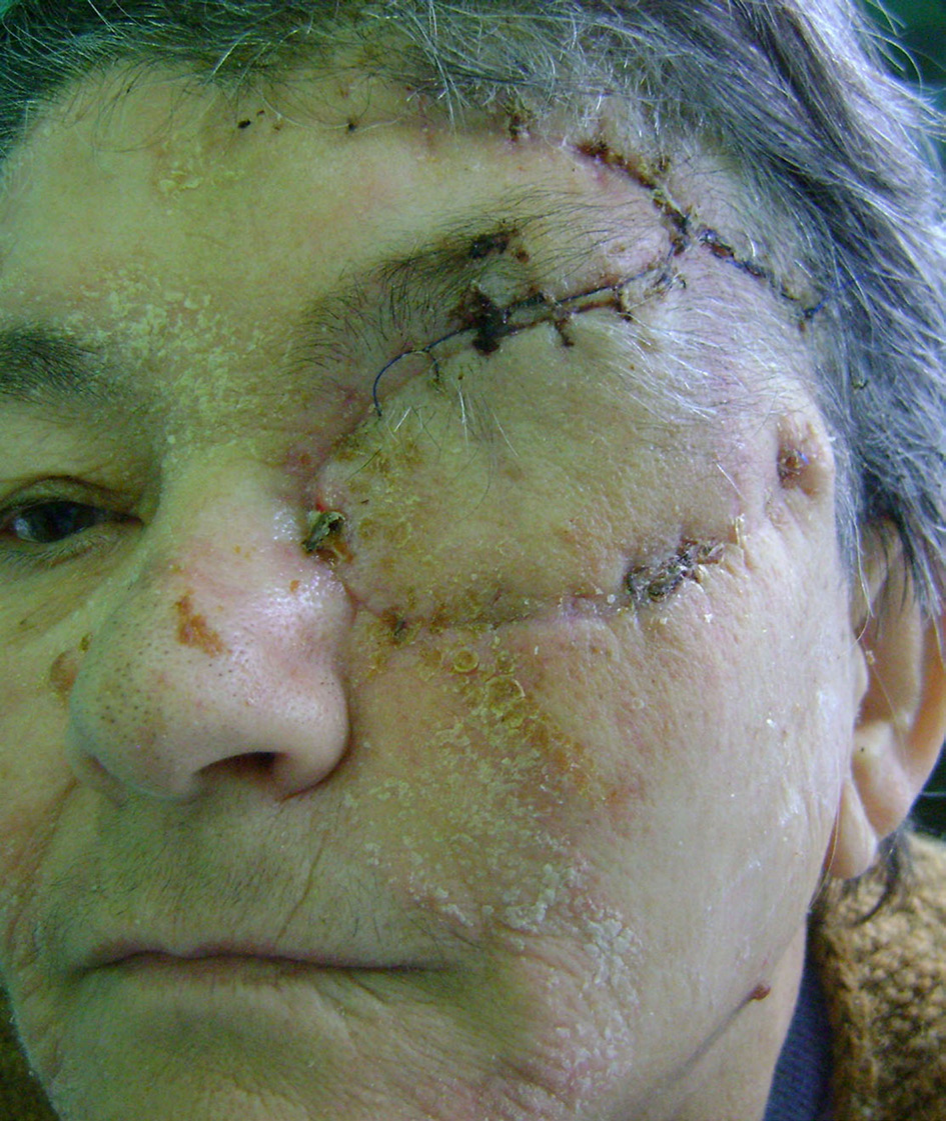
Primarily closed definitive result donor site in the frontal region.

**Figure 4:**
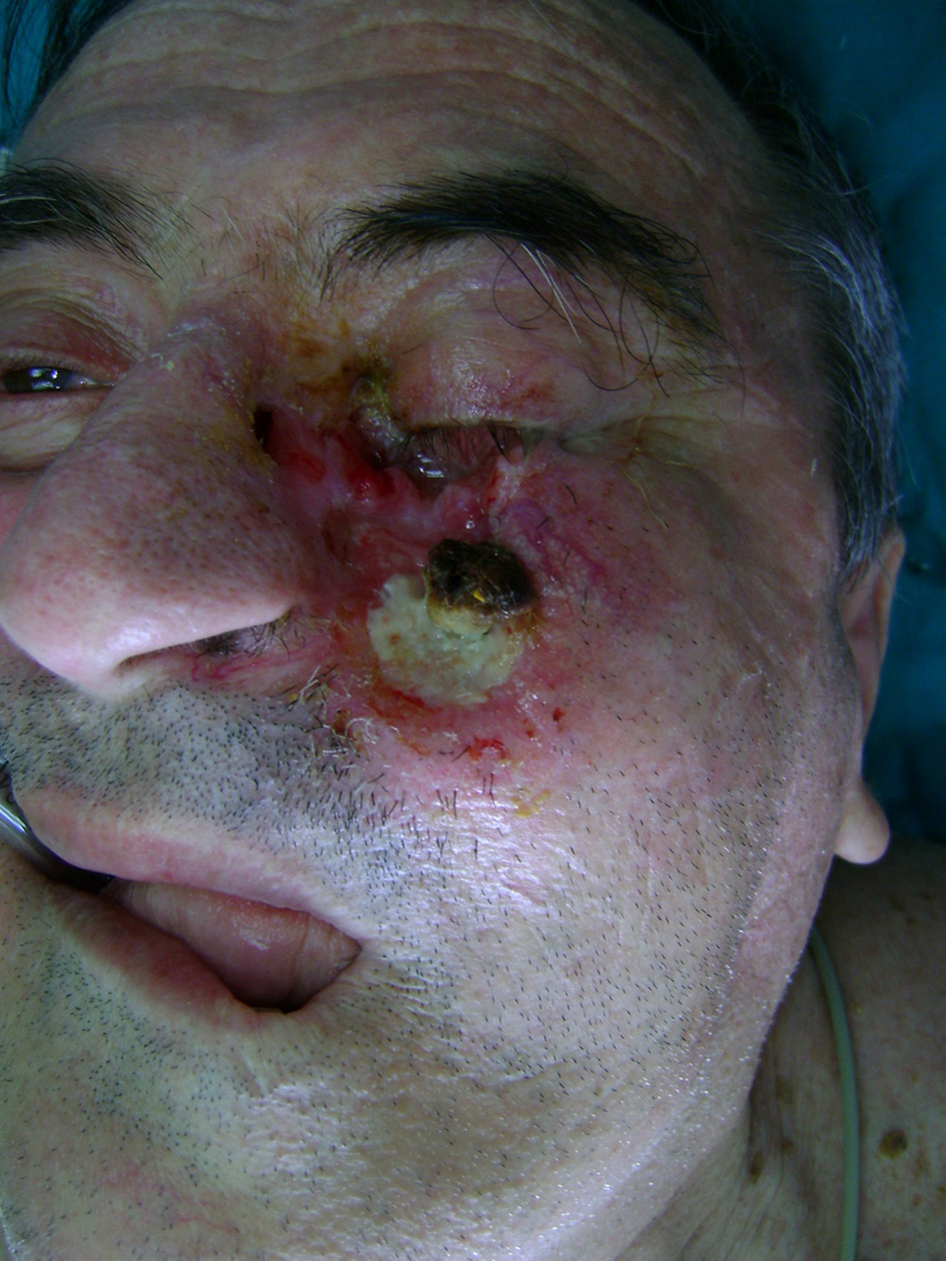
Basal cell skin cancer of the left cheek infiltrating the facial massive.

**Figure 5:**
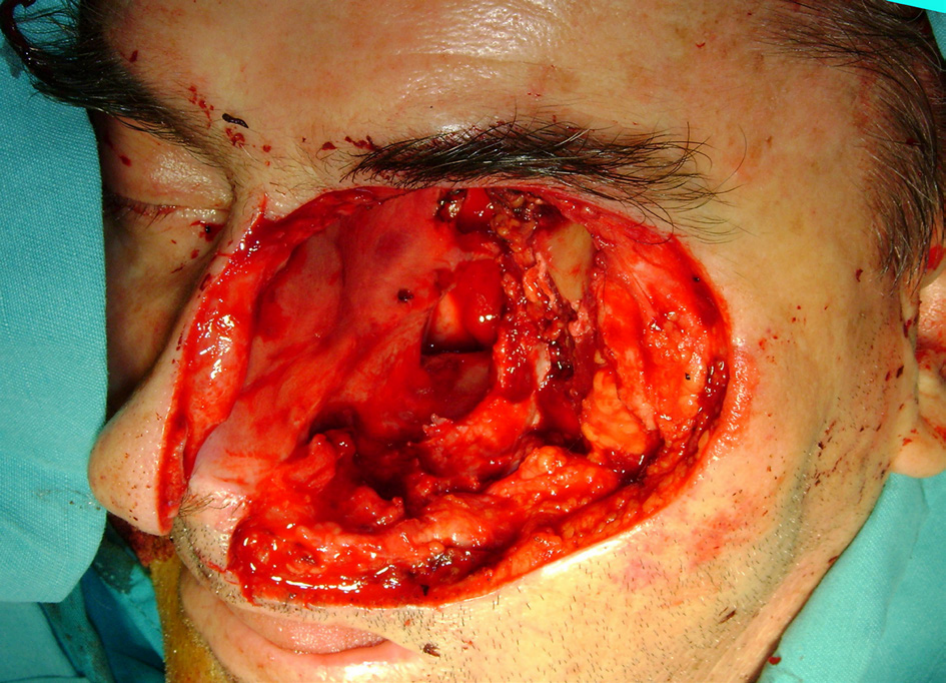
Operative defect including OE, ethmoidectomy, partial maxillectomy, and malar bone resection

**Figure 6:**
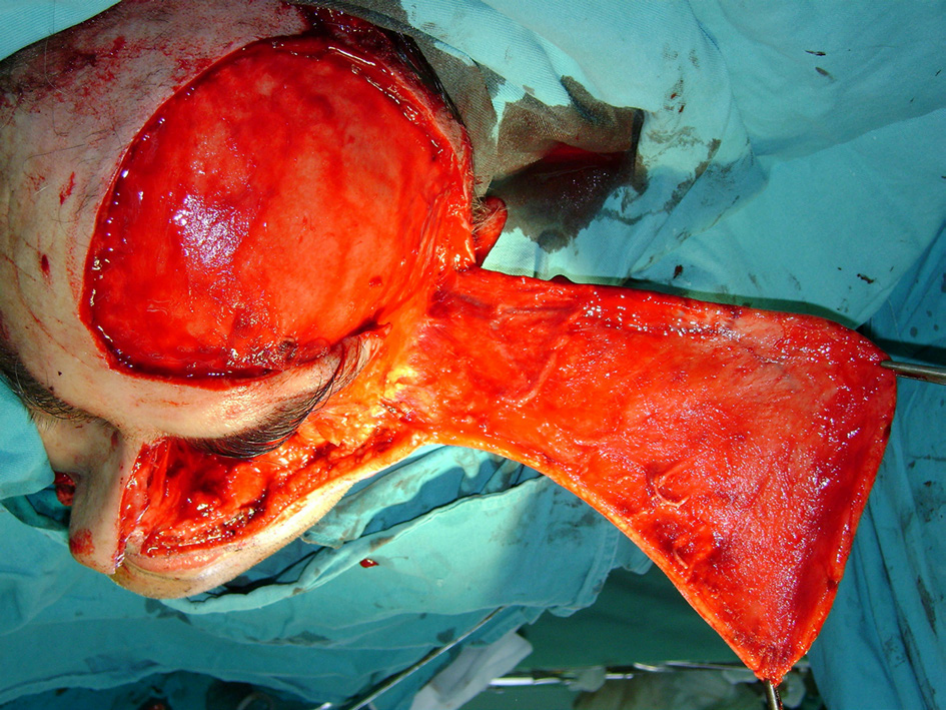
The designed extensive galeal-cutaneous flap.

**Figure 7:**
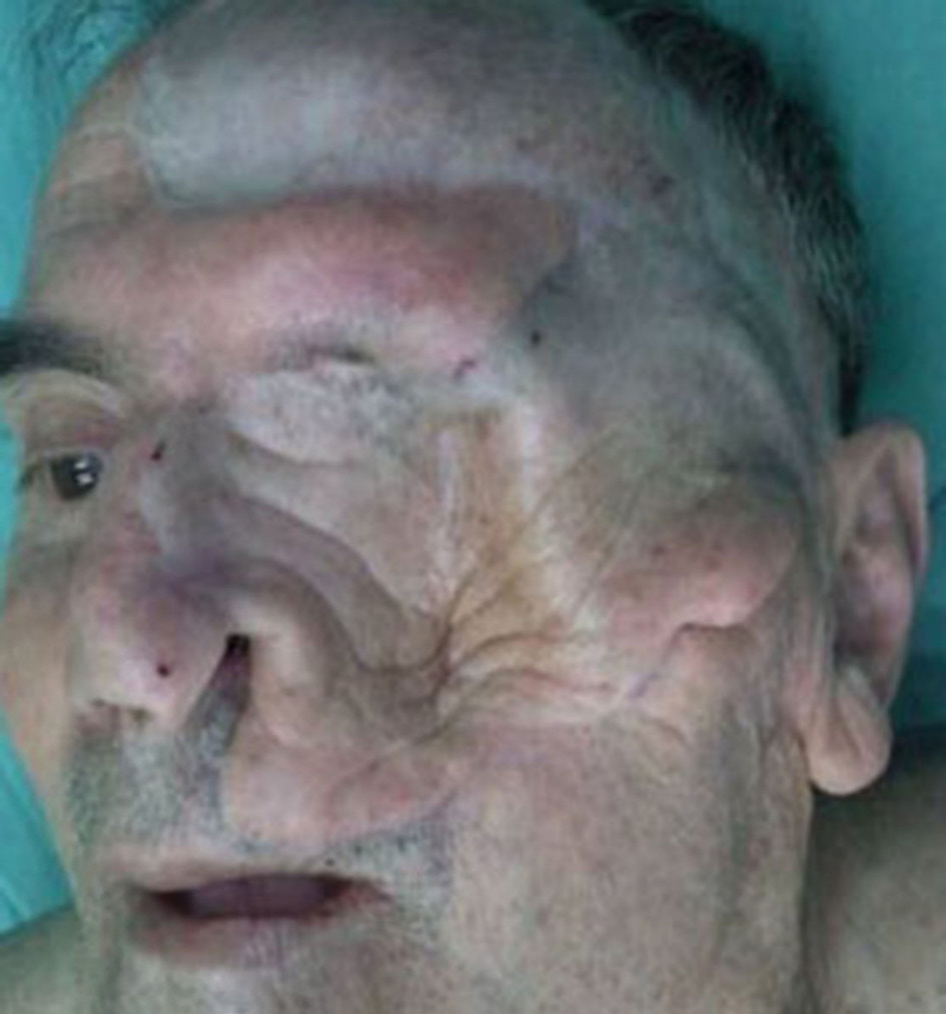
Definitive result. Skin-grafted donor region in the frontal area.

Early postoperative complications were minor, and did not require further surgery. The overall complication rate was 11.53% as follows: hematoma formation in the orbit was drained spontaneously in 1 patient (3.85%) and in 2 patients (7.7%), partial loss of the skin graft appeared and healed by secondary intention. The mean operative time was 114 ± 15 min. The average hospital stay was 7.2 ± 1.1 days. None of the patients stayed in the intensive care unit (ICU). The average length of follow-up from surgery to the last contact or to death was 27.5 ± 13.2 months (ranged 8 to 78 months). Over the long term, the flap molded itself to the orbital cavity. The skin graft over the donor area presented total integration. The aesthetic result was good in all of the patients. According to the decision of the oncological board, all of the patients with skin SCC were irradiated, as well as 2 skin BCC patients with R1 resection (microscopically evident residual tumor on the resection margin). During the follow-up, recurrent tumors were registered in 5 (19.23%) patients. In 2 patients, recurrent tumors were detected as resectable, and they underwent surgery and in a later follow-up, did not present disease progression. In 3 patients, recurrent tumors invaded the skull base, and were unresectable (death in those 3 patients was related to tumor progression). In 7 patients, death was caused by another disease. A total of 16 patients survived the follow-up period. Aesthetic results were considered satisfactory in all of the patients. The clinical cases are shown in Figures 1–7.

## 4. Discussion

OE is one of the most disfiguring procedures in the head and neck region, and some researchers have defined it as one of the most destructive and unpleasant surgeries to perform [8]. Periorbital skin carcinoma is very common, and due to the anatomical features of the eyelids and periorbital region, patients frequently present with advanced tumors. When tumor penetration through orbital septum and involvement of the orbit bone walls are seen on CT scans, OE is required. The results of the current study were consistent with other series where the main indication for the exenteration was skin carcinoma (periorbital or eyelid). Periorbital skin malignancies invading the orbit and bony structures could be defined as neglected cases [9]. In the present study, a series of 26 patients was presented, dominantly of male sex (M:F = 14:12). Male sex was also dominant in other series [8,10–16]; however, some studies have referred to female predominance [9,17]. The average age of the patients herein was 75.69 years. The average age of the patients in other studies also indicated the elderly population, ranging from 60 to 77 years, and these results correlated with the data herein [1,9,11,12,14–16,18]. The primary tumor sites in the current study were medial cantus (50%) and lower lid (34.62%). The study of BCC with orbital invasion by Sun showed the same data [1]. Some researchers have found a higher incidence of lower lid cancers invading the orbit [13,19]. Skin BCC is the most common in the periorbital region and despite its slow progression, it may invade the orbital structures [9]. The most frequent histological type in the current series was BCC. BCC most commonly affects the lower eyelid and medial canthus, which was in correlation with the current results [3,10,11,14,20]. However, an equal incidence of BCC and SCC has also been reported in some studies [15]. SCC is associated with additional risk of perineural spread along the branches of the trigeminal nerve [9]. Some studies have reported a predominance of SCC [15,21]. OE is not reserved only for primary orbital tumors, but is often required for periorbital skin tumors that invade the orbit. Invasion of the fat and muscles of the orbital apex and infiltration of the conjunctiva or sclera are absolute indications for OE [22]. In the present study, OE was performed in 57.7% of the patients for recurrent tumor and secondary orbital spread after the primary treatment of skin cancer, and in 42.3% of the patients for primary advanced skin cancers with evident orbital invasion upon first presentation. Secondary orbital tumors were also indicated as the main indications in other studies [11,15,19]. Although radical surgery is the primary goal, in most of the series, clear margins were not achieved in all of the patients [12]. In the current series, clear margins were recorded in 20 of the 26 patients (76.92%), while in other reports, clear margins ranged from 33% [9] and 50% [19] to 61% [13,21]. Some studies have also reported perineural invasion as the reason for inadequate radicality at a rate of 63% [16]. In the present study, total and extended OE were almost equally performed (53.85% vs. 46.15%). This finding was in agreement with that reported by Nasab [14]. In the series of Duman [11], total exenteration was required in 75% of the patients, while extended exenteration was required in 25%, while Croce [8] reported 7 extended exenterations and 1 total exenteration in his series.

In the present series, only 1 case of extended OE included the ethmoid, maxilla, and malar bone, while in 6 cases, only the medial wall was resected (ethmoidectomy). In 3 cases, the inferior and lateral orbital walls were resected (superstructure maxillectomy and malar bone resection), and in 2 cases, resection of the superior and lateral orbital wall was indicated. The aim of reconstruction is to establish contour symmetry with the opposite side of the face and it is important that the flap covers the line of the orbit [9,10]. The current study presented the role of the lateral frontal galeal-cutaneous flap in primary reconstruction after OE. Numerous techniques have been described for reconstruction of the exenterated orbit [3]. Spontaneous granulation with epithelization was one of the first techniques reported. In 1 report with 25 cases, in which the orbit was left to granulate, all of the patients presented with tumor progression/recurrence on follow-up, and the researchers suggested the use of the orbital granulation approach when the tumor clearance was not certain [17]. However, secondary granulation with epithelization is rarely applicable, as it results in serious discomfort and delays postoperative irradiation. Primary reconstruction promotes healing and provides a better aesthetic appearance [22]. The average healing time for spontaneous granulation is 14 weeks to 6 months [22,23]. Primary closure could be obtained by cheek advancement [24]. Local options include split thickness skin graft [14], or a full thickness skin graft [8]. Skin grafts for reconstruction could be used for defects without bony wall resection [2]. In cases where OE is combined with resection of the orbital walls, the usage of flaps is advised for more extensive defects. The aim of the flap is to isolate the orbit from the nasal cavity and paranasal sinuses, prevent fistula, and protect the cranial contents from exposure in extensive defects. A frontal flap provides good color and texture, similar to the rest of the facial skin, and represents an excellent single-stage reconstruction. The use of local flaps is recognized as advantageous with regards to spontaneous healing of the skin grafting, especially in anticoagulated patients. The use of local flaps reduces hospital stay and allows earlier social reintegration of the patient [10]. The predominant usage of a temporalis muscle flap with skin graft has also been reported previously [13,22]. A temporalis muscle pedicled flap has a robust vascular supply and is preferred to transpose the orbit through a window in the lateral orbital wall (transorbital) [9]. A galeal fascial or pericranial flap based on the supratrochlear artery has been described [8,25]. Croce [8] used a lateral-based frontal fasciocutaneous pedicle flap and full thickness skin graft in the oldest patient in his report. The disadvantage of a temporalis muscle flap is the visible hollowness of the temporal area, even though none of patients expressed concerns about that [9]. A bilobed forehead flap based on the supraorbital and supratrochlear pedicle could be used as a local flap in orbital reconstruction [26]. Some researchers have described the use of a cervicofacial rotation advancement flap for orbital reconstruction [3], while others have referred to the use of pedicle muscle flaps as pectoralis major flaps [27] or latissimus dorsi muscle flaps [8]. In the present series, a temporalis muscle flap was also performed in 2 patients, where the medial half of the orbital roof was resected in order to separate the cranial structures with a sufficient mass of muscle.

In cases where the orbital cavity is not completely filled with transferred tissue, the residual scaring can displace the eyebrow downward and lead to facial asymmetry. Hannasono [2] defined such reconstructions as open cavity reconstructions, wherein bulky flaps, such as rectus abdominis, completely fill the orbital cavity, which were defined as closed orbital reconstructions [2]. The contracture is less intensive and there is no displacement of the surrounding tissues. However, the patient cannot wear orbital prothesis. In the reconstructions herein, the flap was bigger than the orbital defect and the problem of eyebrow displacement was not encountered. Some researchers have suggested the use of a microvascular flap for orbitomaxillary defects as the best solution [28]. Microsurgical flaps are time-consuming and require the good general status of the patient. OE is usually performed in older patients with significant comorbidities. These increase the surgical risk and may worsen the postoperative outcome [29]. Free flaps extend the duration of surgery and an additional team is needed [9,22]. Prolonged surgery with free flap reconstruction may increase the risk of postoperative ICU requirements and complication rates. Free flaps should be reserved for selected cases. For orbital reconstruction, some researchers have used microvascular dorsalis pedis, flaps [30], or free myocutaneous gracilis flaps [31]. The flap used in the present study consisted of galea and skin of the frontal region, avoiding the use of skin grafts for the orbital walls or for the coverage of a previously used galea flap alone. The donor region in the frontal area was able to be closed primary in most of the patients or skin grafted. The transferred flap was resistant enough to radiation therapy and no radiodermatitis was registered. In 2 patients temporalis major muscle was also used in order to separate the intracranial contents from the exterior environment. The advantages of the lateral frontal galeal cutaneous flap include its easy design and elevation, low donor area morbidity, adequate cutaneous covering of the orbital area, possibility of the closure of communication with the paranasal sinuses, and preservation of the temporal muscle. The donor region was primary closed in 17 patients, but in 7 patients, it was covered using a partial thickness skin graft. 

The average operative time was 114 min, which was short when compared to the reported much longer time (up to 500 min) using free microvascular flaps [9]. Based on the experience gained from this, and other studies, it could be concluded that the use of local flaps is time-saving reconstruction [10]. None of the patients in the current study stayed in the ICU, which was in agreement with the results of Hanasono [2] in cases of orbital reconstruction using a skin graft and local flaps. The average hospital stay herein was 7.2 days, which was in accordance with some studies [2], but inconsistent with others, with a reported longer length of hospitalization that ranged from 7 to 35 days [9,14,15]. We think that OE is indicated most predominantly in older patients with serious comorbidities. The intention is to achieve radical tumor ablation and sufficient reconstruction, and the surgeon should avoid long surgeries using microsurgical free flaps. Early postoperative complications were minor in the current series, and did not require further surgery. The overall complication rate was 11.53% and included a hematoma formation in the orbit that drained spontaneously in 1 patient (3.85%) and in 2 patients (7.7%), partial loss of a skin graft in the frontal region (donor site) was encountered, which healed by secondary intention. As there were no flap-related complications, the flap exhibited the best results, and therefore, came to be the preferred method in our clinic. In the study of Croce [8], all of the patients recovered satisfactorily, but in the series reported by Rabey [3], 2 of the 12 patients required further procedures due to complications from the reconstruction used. Partial necrosis of the flap in 3 patients was reported in another study [13]. In the literature, sinoorbital fistula was the most common complication of the exenteration; however, in the present study, neither sinoorbital fistula nor fungal infection of the orbital cavity was detected, despite the fact that the orbit was left empty in most of the cases. In some reports, intraoperative cerebrospinal fluid leakage was managed with tissue adhesive, without any complications [11]. On the other hand, Shieh [32] reported a case of tension pneumocephalus after OE, wherein their patient was treated with collagen, and an engineered dural substitute fat graft and temporalis muscle flap in order to close the dural tear in the orbital roof. No cerebrospinal leakage or complicated sinoorbital fistula were encountered in the present study. The average length of follow-up from surgery to the last contact or to death was 27.5 ± 13.2 months (range: 8 to 78 months), while Gerring [24] calculated survival after a mean follow-up of 17.5 months. Pain and swelling were the most common presenting features of patients with recurrent tumors [15]. The incidence of recurrent tumor in the current study was 19.23% (5 patients). In 2 patients, recurrent tumor was detected as resectable; hence, the patients underwent surgery and in the follow-up, did not present with disease progression. In 3 patients, recurrent tumors invaded the skull base, and were unresectable (the deaths of those 3 patients were related to tumor progression). The local recurrence was reported as 18.7% [11], 25.8% [16], and 28.5% [19]. In another study, 3 cases of recurrence were detected at an average onset of 28 months (range: 12 to 60) with contralateral exenteration indicated for 1 patient [13]. We support the statement that the survival rate is acceptable enough to justify the undertaking of such a disfiguring and dysfunctional operative procedure [16].

In conclusion, it should be pointed out that the surgical team must be familiar with basic anatomy, as well as have adequate surgical skills for the reconstruction. From the authors’ point of view, galeal-cutaneous flap based on the temporalis superficialis artery is the technique of choice for reconstruction following exenteration and extended OE. Temporalis muscle flap reconstruction is indicated only in cases of extended OE with anterior skull base resection (upper orbital wall), in addition to the galeal-cutaneous flap described in this study. The lateral frontal flap can be considered as an option for patients with contraindication to a microsurgical flap, as one of the simplest procedures in the elderly population with significant morbidities. 

## Author contributions

The authors declare that they have all participated in the design, execution, and analysis of the paper, and they have approved the final version. 

## Informed consent

The study protocol received institutional review board approval (Faculty of Medicine University Nis, Serbia). All of the participants provided the informed consent required, and all of the procedures performed in study were in accordance with the ethical standards of the Institutional and National Research Committee and the 1964 Helsinki Declaration and its later amendments or comparable ethical standards.
